# Small-Scale Perception in Medical Body Area Networks

**DOI:** 10.1109/JTEHM.2019.2951670

**Published:** 2019-11-06

**Authors:** Dou Fan, Aifeng Ren, Nan Zhao, Daniyal Haider, Xiaodong Yang, Jie Tian

**Affiliations:** 1 School of Electronic EngineeringXidian University 47905 Xi’an 710071 China; 2 School of Life Science and TechnologyXidian University 47905 Xi’an 710126 China; 3 Institute of AutomationChinese Academy of Sciences Beijing 100190 China

**Keywords:** Breathing patterns, C-band sensing technique, non-invasive detection, respiratory rate

## Abstract

Objective: Non-invasive respiration detection methods are of great value to healthcare applications and disease diagnosis with their advantages of minimizing the patient’s physical burden and lessen the requirement of active cooperation of the subject. This method avoids extra preparations, reduces environmental constraints, and strengthens the possibility of real-time respiratory detection. Furthermore, identifying abnormal breathing patterns in real-time is necessary for the diagnosis and monitoring of possible respiratory disorders. Method: A non-invasive method for detecting multiple breathing patterns using C-band sensing technique is presented, which is used for identifying different breathing patterns in addition to extract respiratory rate. We first evaluate the feasibility of this non-contact method in measuring different breathing patterns. Then, we detect several abnormal breathing patterns associated with certain respiratory disorders at real time using C-band sensing technique in indoor environment. Results: Mean square error (MSE) and correlation coefficient (CC) are used to evaluate the correlation between C-band sensing technique and contact respiratory sensor. The results show that all the MSE are less than 0.6 and all CC are more than 0.8, yielding a significant correlation between the two used for detecting each breathing pattern. Clinical Impact: C-band sensing technique is not only used to determine respiratory rates but also to identify breathing patterns, regarding as a preferred noncontact alternative approach to the traditional contact sensing methods. C-band sensing technique also provides a basis for the non-invasive detection of certain respiratory disorders.

## Graphical abstract

Normal breath for one minute.

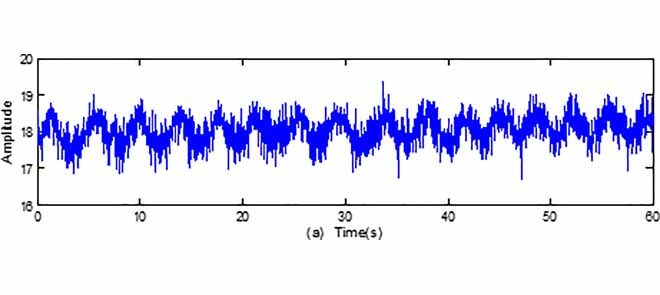

## I. Introduction

**FIGURE 1.**
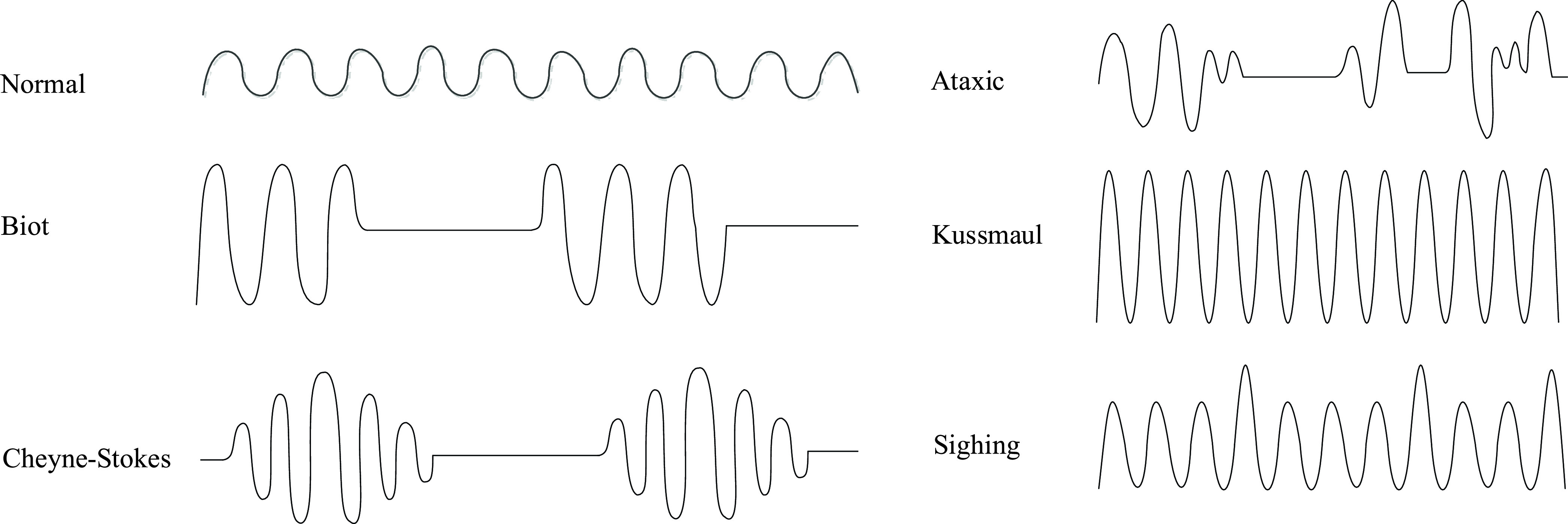
Respiration patterns of normal and abnormal.

BReathing is not just a matter of inhaling the air and exhaling the air. The entire respiratory pattern is important to human health. Rate, depth, timing, and consistency of breaths are all vital to the delicate balance of respiration and metabolism. On the one hand, the respiration rate is one of the four primary vital signs of life, which is useful in detecting or monitoring medical problems [1]. On the other hand, certain diseases or injuries can cause change in the breathing pattern. So careful observation of the respiratory rate and pattern is a crucial part in the diagnosis and during the course of treatment of various diseases [2]. Normal respiration rate for a healthy adult at rest varies form 12–20 breaths/min and it is considered abnormal to have a rate under 12 breaths/min or over 20 breaths/min [3]. Abnormal breathing patterns indicate the potential for injury or metabolic disorders. For example, Biot’s respiration is caused by damage to the pons due to strokes or trauma or by pressure on the pons due to uncial or tentorial herniation. Biot’s respiratory pattern can also be induced by opioid use [4]. There are multiple types of abnormal breathing patterns, includes Biot’s respiration, Cheyne-Stokes respiration, Kussmaul breathing, Ataxic breathing, sighing breathing and so on [4]–[6]. Some breathing patterns are presented in Figure 1. The characteristics of these breathing patterns are described clearly in this figure. So the long-term and real-time detection of respiratory signals can be used in the discovery and diagnosis of respiratory disorders. Therefore, there is a need for a non-invasive method which accurately captures respiratory function under various breathing conditions in the area related to respiratory physiology.

Many different measurement methods can be used to obtain the respiration information. The finest monitoring technique is spirometry, which directly measures the volume and flow of air that can be inhaled and exhaled [7]. Some common methods for continuous respiration monitoring in hospital and clinical settings are inductance pneumography [8], electrical impedance pneumography (EIP) [9], and capnography [10]. However, these methods require a patient to visit a hospital, which cause the inconvenience to the patient. Other methods like utilizing pressure sensor arrays [11] or cameras [12] are also used for monitoring respiration. However, there are expensive and light-limited disadvantages to these methods.

Radio Frequency (RF) based monitoring methods overcome above-mentioned drawbacks, and have caught much attention as the most promising candidates. Under this category, these methods can be classified, based on special wireless devices and based on commercial off-the-shelf transmitter-receiver. In these methods special wireless devices are used such as the Doppler radar [13], [14], the ultra-wide-band (UWB) radar [15], [16], and the Frequency Modulated Continuous Wave (FMCW) radar [17], [18]. However, these systems require specialized devices with high complexity, hindering the development of them.

On the other hand, some systems based on commercial off-the-shelf transmitter-receiver are built on the existing wireless network infrastructure. For example, Patwari *et al.* [19] and Kaltiokallio *et al.* [20] utilized the received signal strength (RSS) to detect human breathing and estimate the breathing rate. In recent studies the RSS measurement obtained from the universal software radio peripheral (USRP) devices is used for respiration detection [21]. However, the RSS has lower detection precision because it cannot characterize multipath propagation. Therefore the abnormal breathing (e.g., sleep apnea) is hard to identify from the RSS data. So can we find a more sensitive wireless signal than the RSS data that various breathing patterns can be detected? The answer is yes, the fine-grained channel information is discovered that is much more sensitive than RSS.

Based on the above, we propose a non-invasive detection method based on radio signals, called C-band sensing technique. It has abilities to sense breathing in an indoor environment by using the propagation of electromagnetic waves. Specifically, we build a pair of prototypes operating at C band to capture respiration information. This system leverages the readily available channel information to detect the slight change caused by breathing. Compared with the RSS, our system utilizes fine-grained channel state information that contains both amplitude and phase information of multiple orthogonal frequency division multiplexing (OFDM) subcarriers.

In this paper, we first analyze the feasibility of using C-band sensing technique in detecting different types of breathing patterns. Next, we leverage one pair of prototypes to detect several abnormal breathing patterns relevant to certain diseases in the door environment. These breathing patterns are professionally playing role according to the medical descriptions given in [4]–[6]. In addition, we show that the correlation between wireless signals obtained from C-band sensing technique and respiratory sensor data for various breathing patterns. Finally, this demonstrates that C-band sensing technique can provide non-contact, continuous fine-grained respiratory patterns detecting. It also supports real-time and long-term respiratory patterns monitoring in home.

The main contributions of this paper are summarized as follows.

• We propose a non-invasive method, C-band sensing technique, which not only can detect the respiratory rate but also can capture different types of breathing patterns.
• Our proposed system utilizes fine-grained channel state information to detect various breathing activities, especially for detecting multiple abnormal breathing patterns. The proposed C-band sensing technique provides a basis for the non-invasive detection of certain respiratory disorders.
• Extensive experiments show that our system has significant correlations with dedicated respiration sensor, in other words, our system can achieve comparable performance as dedicated respiration sensor.

The rest of this paper is organized as follows. Section II presents the theoretical foundation of C-band sensing technique in detecting respiratory activities. Section III introduces the overall design of the proposed system that covers feasibility analysis and system overview. Section IV describes the signal processing used in wireless data and reference data. Section V describes the experimental setup and discusses the experimental results for various breathing patterns. Finally, conclusion is given in Section VI.

## II. Methods and Procedures

The proposed method uses C-band sensing technique to detect breathing, and this technique can sense the minute movements by collecting fine-grained channel state information through propagation of electromagnetic waves. In the indoor environment, the RF signal generated by the transmitter reaches the receiver through multiple paths, thus forming the receiving signal with multipath superposition. This receiving signal carries information, reflecting environmental characteristics under the influence of the propagating physical space. The environment refers to the physical space of signal transmission, including human factors (human position, breathing, etc.) and environmental factors [22]. RF based sensing method uses precisely the influence of physical space on the signal to inversely deduce the characteristics of the sensing target to realize sensing.

**FIGURE 2.**
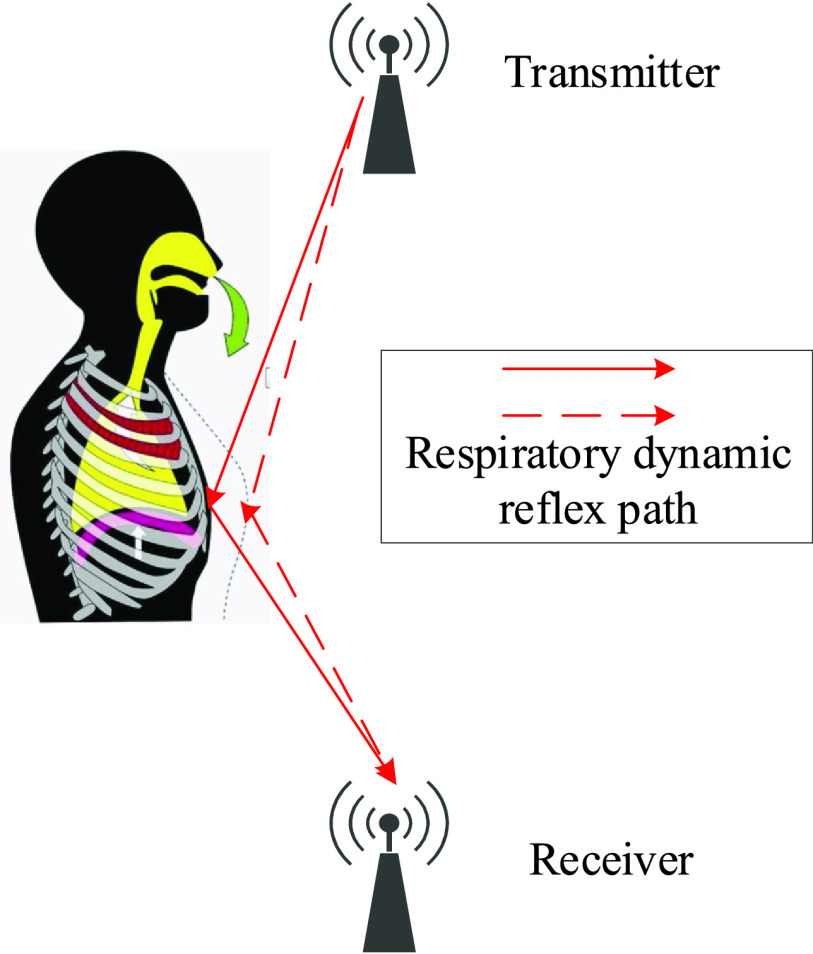
The mechanism of detecting breathing by C-band sensing technique.

When a person exists in the physical space, the additional path is introduced due to the body’s reflection or diffraction of signals. Therefore, the influence of human behavior on the propagation of signals, as part of the physical channel, is also recorded by the receiving signals and described in the form of channel state information. For this experiment, the transmitter continuously transmits wireless signals with a specific frequency, and the receiver receives signals sent by the transmitter. Meanwhile the minute changes in the chest and abdomen caused by breathing, induces change in the signal propagation path (as shown in Figure 2), recorded by the received signals in the form of channel sate information.

C-band sensing technique adopts OFDM technology which divides the single spatial stream into a series of orthogonal channels, called subcarriers. Each received channel state information packet is composed of a group of 30 subcarriers, given as follows:

\begin{equation*} H=[H \left ({f_{1} }\right), H\left ({f_{2}
            }\right), H\left ({f_{3} }\right),\ldots, H \left ({f_{n}
            }\right)]\tag{1}\end{equation*}

here $H $ represents the Channel Frequency Response (CFR), $n=30$ is the total number of subcarriers.

Each subcarrier contains amplitude and phase information. Assuming $\text {k}\in [{1,30}]$ is the sequence number of the subcarrier, the CFR of the kth subcarrier can be expressed as:

\begin{equation*} H(f_{k})=\vert \vert H(f_{k})\vert \vert
            e^{j\angle H(f_{k})}\tag{2}\end{equation*}

where, $\vert \vert H \left ({f_{k} }\right)\mathrm {\vert \vert }$ represents the amplitude information and $\angle H\left ({f_{k} }\right)$ denotes the corresponding phase information.

To detect breathing activities, the data packets need to be collected continuously within certain time, and all the recorded measurements for time duration is represented as:

\begin{equation*} \boldsymbol {H}=[H^{1}, H^{2},
            H^{3},\ldots,H^{N}]\tag{3}\end{equation*}

$N$ is the total number of data packets (CFR) received and serve as the primary input for detecting and analyzing breathing patterns.

**FIGURE 3.**
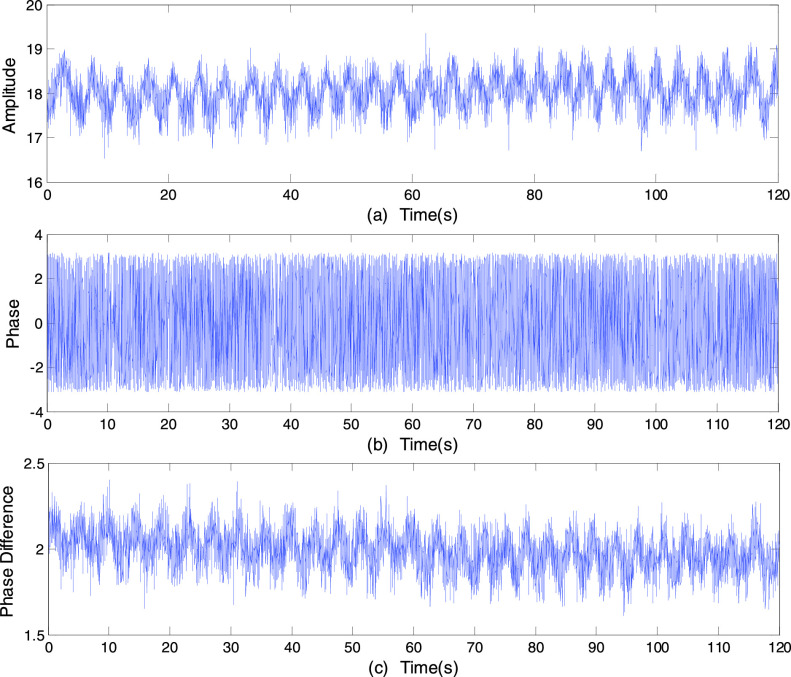
The subcarrier sequence collected over a period of time when a person sits in a chair quietly. (a) The original amplitude information. (b) The original phase information. (c) The original phase difference.

Figure 3(a) and 3(b) shows the original amplitude and phase information of a subcarrier using data collected over a period of time when a person sits in a chair quietly. The sitting posture is selected to detect breathing activity because breathing rate has little change while sitting [23]. An obvious periodic up and down trend can be observed from the amplitude information, which could be caused by the person’s breathing. But the phase information is random and cannot be used directly. Therefore, the processing procedure is essential.

To obtain the available phase information from the raw channel state information, the phase difference between two receiving antennas is used to sense human motion [24]. The key idea is that the phase difference profile for the stationary states is stable enough to distinguish motions. So can the phase difference between two receiving antennas detect respiration? A sinusoidal like periodic wave can be seen clearly from the phase difference between two receiving antennas as shown in Figure 3(c). We adopt one transmitting and three receiving antennas configuration in this experiment. Moreover, we note that the amplitude information presents a better performance than the phase difference. Therefore, the amplitude information is used for breathing patterns detection in the next section.

## III. System Design

In this section, we first validate the feasibility of detecting different breathing patterns using C-band sensing technique and lately we presented the overview of our system design.

### A. Feasibility Analysis

Based on the previous research work [23], the proposed C-band sensing technique can detect normal breathing activity, but it is not known if this method can detect abnormal breathing activity such as apnea. Therefore, before detecting different breathing patterns, we need to know whether our proposed method can detect breathing rate, depth and pause. For this purpose, we perform several different experiments to investigate the feasibility of C-band sensing technique on detecting different breathing patterns. In this experiment, the subject is requested to:

(1) Breathe normally for one minute;
(2) Breathe normally followed by deep breaths;
(3) Breathe deeply, pause, and then normal breathing.

**FIGURE 4.**
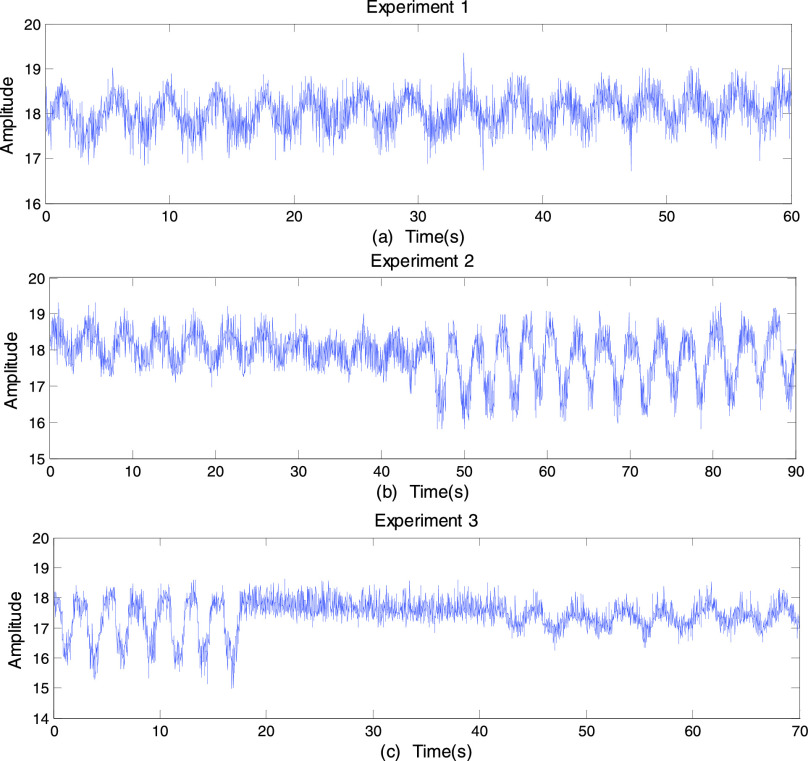
(a) Experiment 1: Normal breaths for one minute. (b) Experiment 2: Normal breaths followed by deep breaths. (c) Experiment 3: Deep breaths, pause followed by normal breaths.

The results of these three experiments are presented in Figure 4(a), 4(b) and 4(c) respectively. The respiratory rate is measured by counting the number of breaths for one minute [2]. It is legible from Figure 4(a) that there are 15 breaths for one minute, with the respiratory rate of 0.25 Hz. From Figure 4(b), the subject first breaths normally 11 times and then takes 13 deep breaths. The amplitude fluctuation of normal breathing is less than 2 values (from 17 to 19) while the amplitude of deep breathing fluctuates more than 2 values or some even by 3 values. An apparent apnea can be observed between 18 and 40 seconds in Figure 4(c). These three experiments illustrate that rate, depth and pause of respiration can be detected perfectly by our proposed method. Based on these findings, there is no doubt that using C-band sensing technique can detect various breathing patterns.

### B. System Overview

**FIGURE 5.**
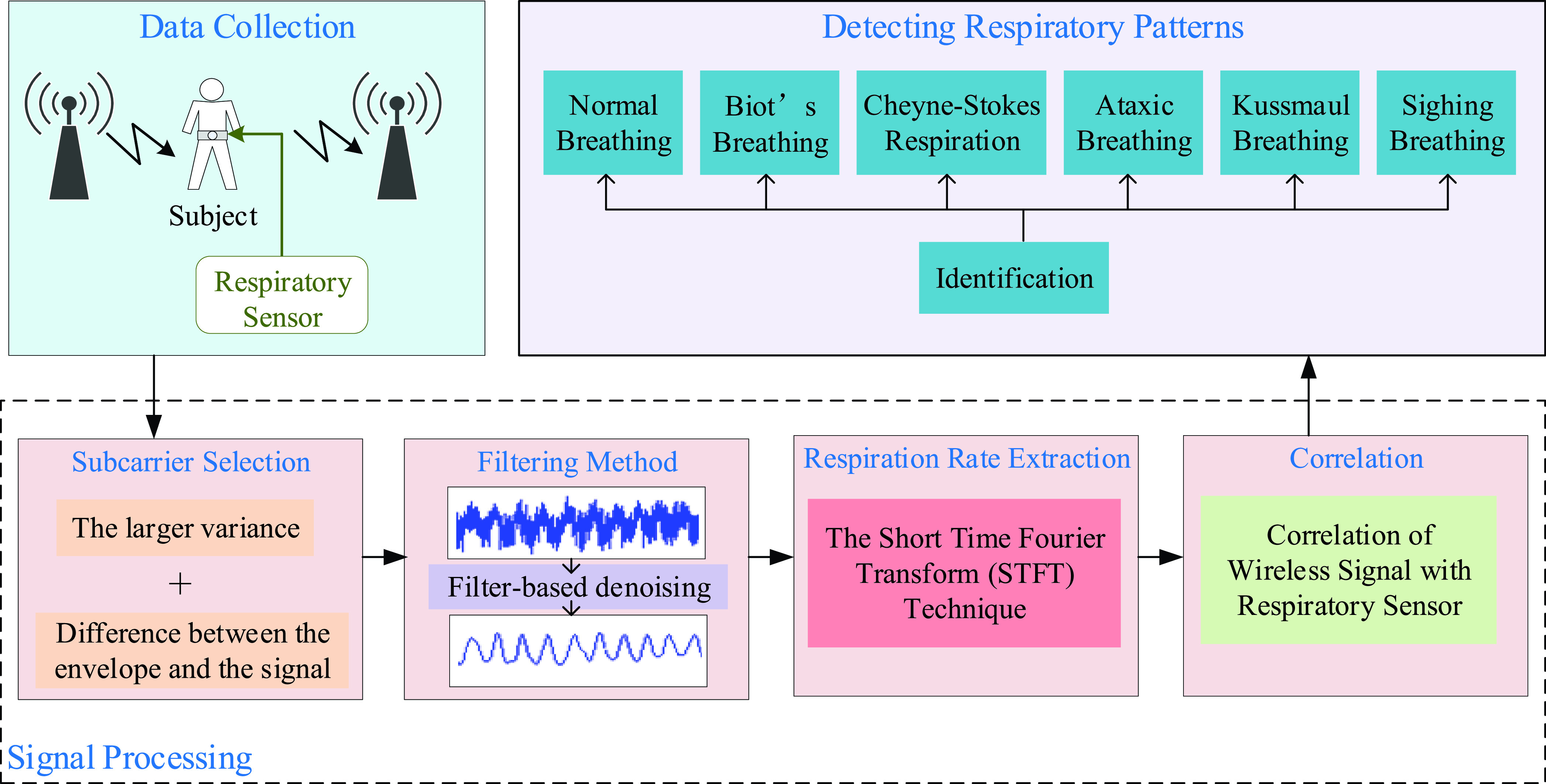
Overview of system flow.

The basic idea of the proposed system is to detect and analyze respiratory patterns through fine-grained channel state information collected by C-band sensing technique. The structure of this system mainly consists of three modules: data collection, signal processing and detecting respiratory patterns, as illustrated in Figure 5.

For data collection, the C-band prototypes are used to collect time-series amplitude measurements that are the primary input of our system. They utilize system-generated periodic traffic to achieve continual long-term data acquisition. These data are then transported to signal processing module and is taken as the input of this module for further analysis. Because there are various abnormal breathing patterns to detect, the data processing method of normal breathing is no longer applicable. So we propose a signal processing module more suitable for multiple breathing patterns. Signal processing module includes four parts; the subcarrier selection method is firstly conducted. The subcarrier selection is of crucial importance because the amplitudes of different subcarriers are expressing different sensitivity to the minute movements caused by breathing. We propose a subcarrier selection method based on scoring mechanism, which selects subcarriers by scoring the variance of subcarriers and the difference between the envelope and the signal. Second, the wavelet filter [25] and the moving average filter [26] are used to denoise and smooth for the selected subcarriers. The third step is to estimate respiratory rate. Spectrogram analysis based on Short Time Fourier Transform (STFT) is used in extracting the respiration rate in each breathing pattern. Finally, the correlation between the two is established by comparing the measurements made using C-band sensing technique with the measurements made by contact respiratory sensor. After these processes, normal breathing and six abnormal breathing associated with certain breathing disorders are detected.

## IV. Signal Processing

In this section, signal processing methods are described in detail. This module contains subcarrier selection method, filtering method, respiratory rate extraction and correlation of wireless signal with respiratory sensor.

### A. Subcarrier Selection Method

**FIGURE 6.**
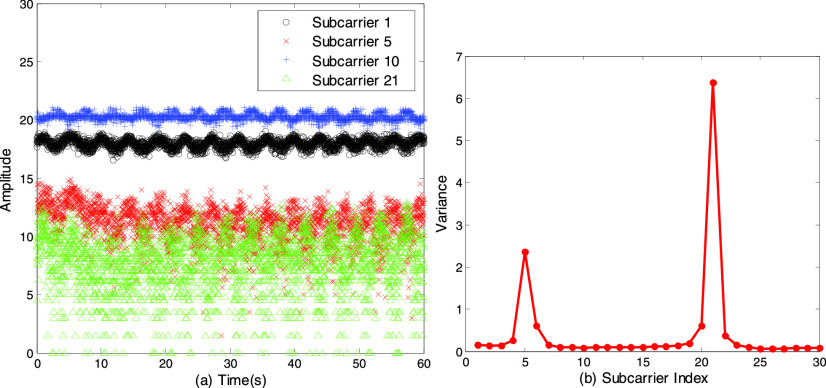
(a) The amplitude information of four subcarriers when a person breathes normally. (b) The variance of 30 subcarriers.

A group of 30 subcarriers can be obtained at the same time from each channel state information. Figure 6(a) shows the amplitude information of four subcarriers (subcarrier 1, 5, 10 and 21) over a period of time when a person breathes normally. We observe that the amplitudes of different subcarriers display different sensitivity to breathing behavior, which is because the frequency of subcarriers is different. For better detection of the breathing activity, it is necessary to remove the subcarriers not sensitive to the breathing activity.

We first consider choosing subcarriers with larger variance. Figure 6(b) represents the variance of 30 subcarriers. As can be seen from Figure 6(b), the variance of subcarriers 21 and 5 is significantly higher than that of other subcarriers. However, from the Figure 6(a) it is evident that subcarriers 21 and 5 fluctuate chaotically and contain more outliers. Therefore, for subcarrier selection, not only the variance but also the difference between the envelope and the signal should be considered. As higher variance means more sensitivity and too much difference between envelope and signal indicates more outliers. By synthesizing these two aspects, we propose a subcarrier selection method based on scoring mechanism. To be specific, each subcarrier is scored in both the variance and the difference between the envelope and the signal. We thus use this method to select subcarriers with a high periodicity level for further analysis.

### B. Filtering Method

**FIGURE 7.**
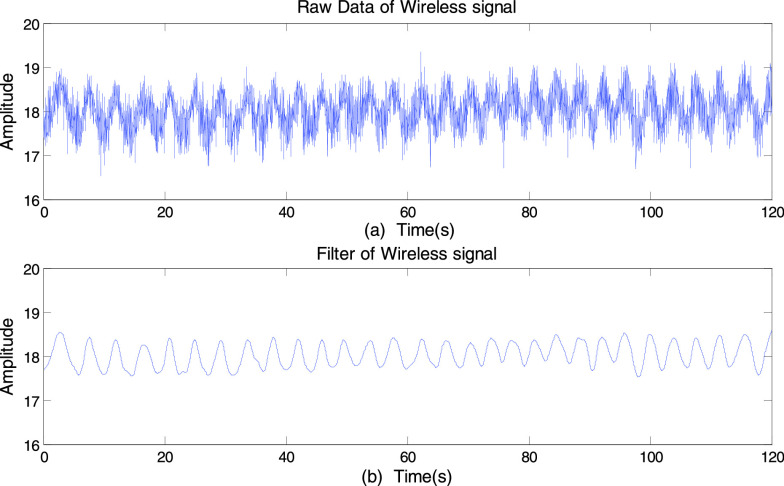
The amplitude information of a subcarrier. (a) Before using the filter. (b) After using the filter.

To improve the reliability of the received data, the noise contained in the received data should be eliminated. Firstly, we used the wavelet filter [25] for denoising, because it can not only filter out outliers but also retain the sharp transitions of signals. Specifically, we apply soft heuristic SURE thresholding and scaled noise option, on detail coefficient obtained from the decomposition of raw data of the selected subcarriers, at level 4 by sym8 wavelet. After that, we further apply a moving average filter to smoothen the data and to remove the high-frequency noise not caused by breathing. The Figure 7(a) and 7(b) represents the amplitude information of a subcarrier before and after using the filter. It can be clearly seen that the sinusoidal waves reflect the periodic up-and-down of the chest and abdomen movements caused by inhaling and exhaling.

### C. Respiratory Rate Extraction

The Short Time Fourier Transform (STFT) technique is used for extracting the respiration rate in each particular breathing pattern. The procedure for computing STFT is to divide a longer time signal into shorter segments of equal length and then compute the Fourier transform separately on each shorter segment. We used the STFT technique to transform the waveforms to spectrograms, so that the amplitude information can be analyzed in the time-frequency domain. The spectrogram uses a sliding window to divide the waveforms into small samples with equal segment, and then executes Fast Fourier Transform (FFT) on these samples. Time, frequency, and FFT amplitude are three dimensions of the spectrogram. The tradeoffs between time and frequency resolution of STFT depends on the window size. The smaller the window, the higher the time resolution will be. However, FFT decreases the accuracy due to the small number of samples, resulting in poor frequency resolution. So it is crucial to choose a suitable window size.

**FIGURE 8.**
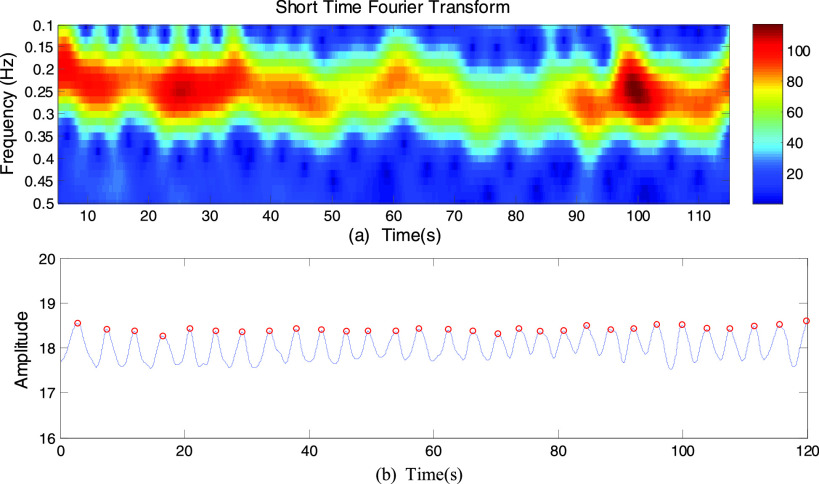
(a) Spectrogram based on the STFT technique for normal breathing. (b) The breathing cycles based on the peak detection method for normal breathing.

In our experiments, the frequency of the measurements for human breathing is less than 1Hz and their changes are in tens of milliseconds. Thus we choose a Hamming window size of 512 samples as the sliding window, the overlapped size of 511 samples in each segment and an FFT size of 3000 samples. The sample frequency is 50 Hz. By computing, it gives suitable time resolution of 20 ms and frequency resolution of 0.017 Hz to capture the minute chest movements caused by breathing. Figure 8 illustrates an example of a spectrogram for normal breathing (The waveform is shown in Figure 7). From Figure 8, the respiration rate estimated using the STFT technique is 0.25 Hz which agrees with the peak detection method where the respiration rate is 15 breaths/min (0.25 Hz).

The peak detection method can only be used to extract the respiration rate under a normal breathing condition. This method is no longer suitable for extracting the respiration rate because of the non-periodic characteristic of abnormal breathing patterns. Therefore, we choose the STFT technique for respiratory rate extraction.

### D. Correlation of Wireless Signal With Respiratory Sensor

It has been demonstrated that the use of C-band sensing technique can detect respiratory rate, depth and pause in the previous sections. To further evaluate the detection accuracy of C-band sensing technique, we compare the measurements captured using C-band sensing technique with the measurements captured by the contact respiratory sensor. Mean square error (MSE) and correlation coefficient (CC) are used to evaluate the correlation between the two.

MSE is a measure of reflecting the degree of difference between the estimated values and what is estimated. The formula for MSE is given as [27]:

\begin{equation*} \mathrm {MSE}=\frac {1}{n}\sum \nolimits
              _{i=1}^{n} {(\hat {X_{i}}-X_{i})}^{2}\tag{4}\end{equation*}

where $\hat {X_{i}}$ is the denoting values of n number of predications. And $X_{i}$ is a vector representing n number of true values.

The correlation coefficient is a numerical measure of some type of correlation that is a statistical relationship between two variables. We choose the Pearson correlation coefficient to compute the correlation, which is a measure of the linear association between two variables [28]. The formula for the Pearson correlation coefficient (denoted as $\rho$) is:

\begin{equation*} \rho _{X,Y}=\frac {cov(X,Y)}{\sigma
              _{X}\sigma _{Y}}\tag{5}\end{equation*}

where (X, Y) denotes a pair of random variables, *cov* is the covariance, and $\sigma _{X}$ and $\sigma _{Y}$ are the standard deviation of X and Y respectively.

## V. Results

### A. Experiment Setup

We conduct extensive experiments to evaluate the performance of C-band sensing technique in detecting different breathing patterns. In order to better set suitable parameters of C-band sensing technique and obtain precise measurement results, we first use RF generator, spectrum analyzer, vector network analyzer, cable and antennas to briefly analyze the microwave distribution situation of the experiment environment. On this basis, we leverage C-band sensing technique to collect wireless data. Specifically, one pair of prototypes equipped with off-the-shelf network adapter is used to obtain the wireless data. One of them connected with one omnidirectional antenna, works as the transmitter, while the other connected with three omnidirectional antennas (say antenna A, antenna B and antenna C) works as the receiver. The transmitter is made up of a USRP device and a computer equipped with off-the-shelf network adapter, and the receiver includes three USRP devices and a computer equipped with off-the-shelf network adapter. The computer equipped with off-the-shelf network adapter is used to stream data to and from USRP devices. Thus, the total number of spatial streams is 3. Each spatial stream provides 30 subcarriers to upper layer users. The transmitter and the receiver operate at 5.32 GHz with a bandwidth of 20 MHz. Compared with the previous work [23], the transmit power has been increased so that it can detect more subtle respiratory changes. The transmit power is −5 dBm. And the sample rate is set to 50 Hz.

The experiments are conducted in a laboratory with dimension $7\,\,\text {m} \times 5\text{m}$. The subject sits on a chair with a relaxed posture and minimum movement from the body. The transmit and receive antennas are placed at the two sides of the chair in the same line of sight with 2m apart, and their height is parallel to the abdomen. In the meantime, the ground-truth respiration is obtained by a contact respiratory sensor attached to the subject’s abdomen. This device is HKH-11C Digital Respiratory Sensor. Its data bits are 8 bits, baud rate is 9600, and the sampling frequency is 50 Hz, which is consistent with the sample rate of the proposed system.

A total of 6 different participants were invited in this experiment and their details are shown in Table 1. All the human subjects participating in this research gave informed consent. Before collecting wireless data these subjects were trained to role play each breathing pattern professionally. First, the characteristics of each breathing pattern were described to all subject according to medical data, and then these subjects were asked to watch medical video of each breathing pattern and to play role of each breathing pattern. Moreover, before formal experiments we conducted some pre-experiments to check the subjects’ performance of each breathing pattern. In this paper, these participants were asked to perform different types of breathing patterns professionally in real time, including:

(1) Normal Breathing
(2) Biot’s Breathing
(3) Cheyne-Stokes Respiration
(4) Ataxic Breathing
(5) Kussmaul Breathing
(6) Sighing Breathing

**TABLE 1:** Details for Six Participants

ID	Gender	Weight (kg)	Height (cm)
1	Male	74	183
2	Male	67	175
3	Male	85	178
4	Female	58	165
5	Female	50	160
6	Female	52	168

For each breathing pattern experiment, 3 data sets are collected from each subject (only one set of observations from subject 3 is shown in this paper where each breathing pattern was imitated well). This is to ensure that C-band sensing technique has a higher level of accuracy in detecting different breathing patterns.

### B. Result

In this section, the detection of each breathing pattern using C-band sensing technique is described in turn. For each condition, the subject was asked to follow a certain breathing pattern, and the wireless data and sensor data were collected simultaneously to investigate the feasibility of using C-band sensing technique in capturing such conditions. Before each experiment commenced the subject was introduced to the characteristics of each breathing pattern and run exercises to simulate such breathing pattern for some time. In this experiment, the 14th subcarrier of antenna C is selected by the subcarrier selection method for the next step of analysis. In the next step we used the filtering method for the selected subcarriers to get cleaner waveforms. After that, we utilized the STFT technique for the respiration rate extraction and the time-frequency analysis, and more information can be obtained from the time-frequency analysis to understand the breathing activity.

Further, in order to evaluate the detection accuracy of using C-band sensing technique, each data received by C-band sensing technique was compared with the standard respiratory sensor measurement as a reference. For this purpose, both the results form C-band sensing technique and respiratory sensor were normalized with a range of [−1, 1], and then MSE and CC were computed by Equation (4) and (5) to find the correlation of the breathing patterns obtained. The formula of normalizing the data to the range of [a, b] is as follow:

\begin{equation*} X^{\prime }=a+\frac
              {X-X_{min}}{X_{max}-X_{min}}(b-a)\tag{6}\end{equation*}

where a = −1 and b = 1.

Table 2 shows the performance evaluation of the results for the above-mentioned six breathing patterns. MSE and CC are used for the validation of the normalized C-band sensing technique measurements in comparison to the normalized respiratory sensor measurements. From Table 2, all the MSE are less than 0.025 and all CC are more than 0.84. The results consistently suggest that C-band sensing technique is highly correlated with the contact breathing sensor.

**TABLE 2:** The Performance Evaluation of C-Band Sensing Technique Measurements Compared to Respiratory Sensor Measurements

Types of Breathing	MSE	CC
Normal Breathing	0.0128	0.9550
Biot’s Breathing	0.0236	0.8698
Cheyne-Stokes Breathing	0.0176	0.8936
Kussmaul Breathing	0.0184	0.8822
Ataxic Breathing	0.0248	0.8417
Sighing Breathing	0.0202	0.9040

#### 1) Normal Breathing

**FIGURE 9.**
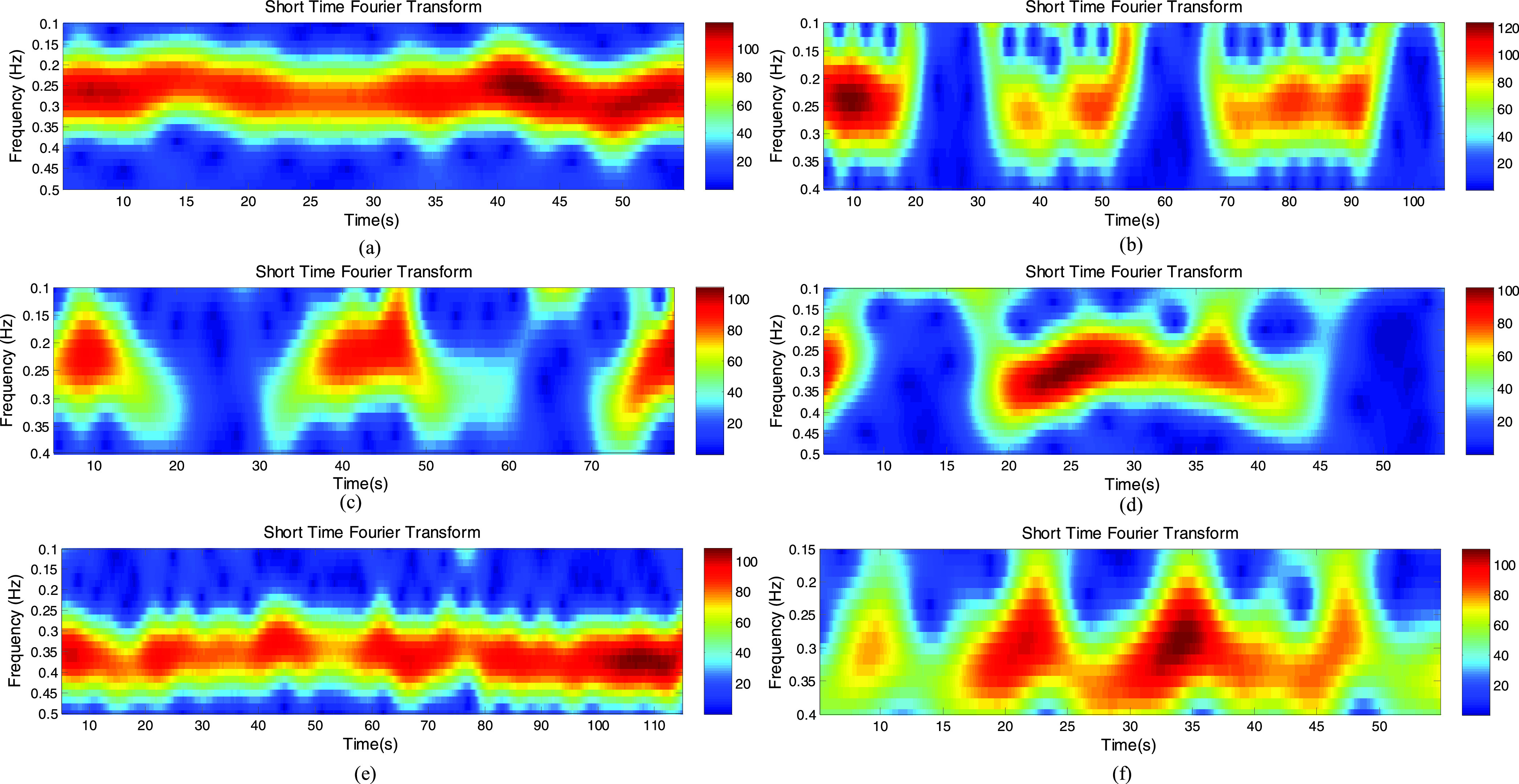
Spectrograms with different types of breathing patterns. (a) Normal breathing. (b) Biot’s breathing. (c) Cheyne-stokes breathing. (d) Ataxic breathing. (e) Kussmaul breathing. (f) Sighing breathing.

**FIGURE 10.**
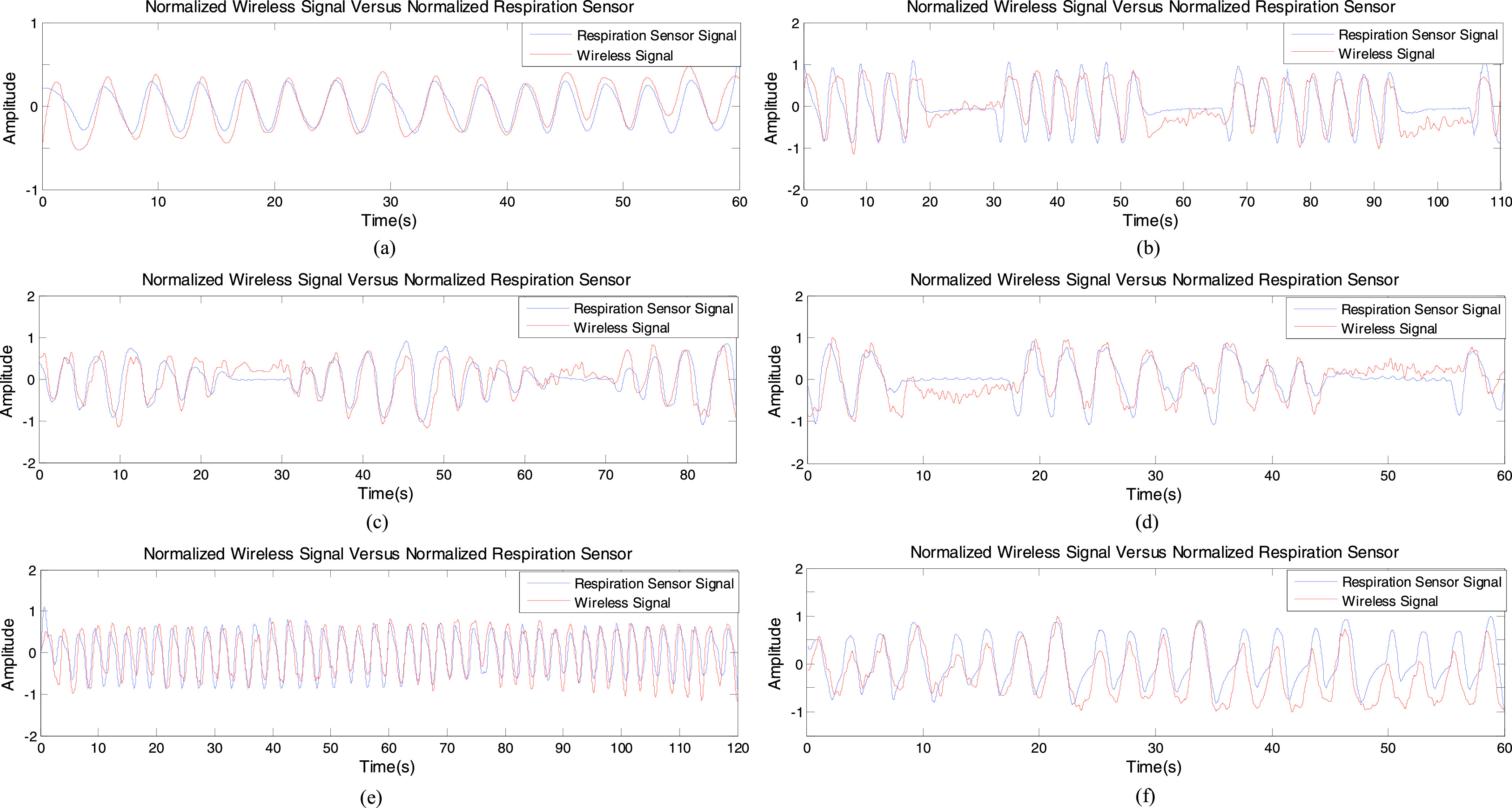
Normalized filtered wireless signal versus normalized respiration sensor signal. (a) Normal breathing. (b) Biot’s breathing. (c) Cheyne-stokes breathing. (d) Ataxic breathing. (e) Kussmaul breathing. (f) Sighing breathing.

Normal breathing is the free and easy respiration when at rest. The normal respiratory rate is 12–20 breaths per minute for adult. For this breathing pattern, the subject was asked to breathe normally and at ease. From Figure 9(a), the estimated respiratory rate is 0.26Hz corresponding to15.6 breaths/min which agrees with the breathing waves as shown in Figure 10(a). And Figure 10(a) shows the normalized wireless signals compared to the normalized respiration sensor signals. The calculated MSE and CC is 0.128 and 0.9550 respectively (see Table 2). This illustrates the significant correlation between the two.

#### 2) Biot’s Breathing

For this experiment, the subject was asked to simulate Biot’s breathing, which is characterized by regular deep respirations interspersed with periods of apnea. Figure 9(b) shows a breathing rate of 0.24 Hz but from Figure 10(b), there are approximately 18.5 breaths in 110 seconds, equivalent to the breathing rate of 0.17 Hz. This indicates that it is not accurate to estimate the respiratory rate of this breathing patterns using time-frequency analysis. Even so, some useful information can be acquired from the spectrogram. From the spectrogram shown in Figure 9(b), we can clearly see that there are 3 pauses in breathing for 20–30 seconds, 55–65 seconds and 95–105 seconds respectively, which is in line with the result shown in Figure 10(b). The spectral analysis is beneficial for detecting the apnea state. The correlation between the normalized wireless signals and the normalized respiration sensor signals in this breathing pattern is shown in Figure 10(b) and Table 2.

#### 3) Cheyne-Stokes Breathing

Cheyne-Stokes breathing demonstrates periods of gradual hyperpnoea alternating with periods of apnea, which have the crescendo-decrescendo pattern. Cheyne-Stokes breathing is a classic breathing pattern seen in individuals with severe neurological or cardiac disease [2]. As for Cheyne-Stokes breathing, from Figure 10(c), there is a periodic breathing change with a crescendo-decrescendo type of sequence followed by an apnea. It can be observed clearly that C-band sensing technique as a non-contact method has ability to detecting the changes of breathing, and gives the good correlation to the respiratory sensor. In the time-frequency analysis, two apneas for 20–30 seconds and 60–70 seconds are detected, shown in Figure 9(c). The breathing rate estimated using the STFT technique (0.22 Hz from Figure 9(c)) is approximately in agreement with the one extracted, using the breathing waveform (0.21 Hz from Figure 10(c)).

#### 4) Ataxic Breathing

Ataxic breathing is characterized by unpredictable irregularity in breathing pattern, breathing may be deep or shallow, slow or rapid and even brief pause. Biot’s respiration caused by damage to the pons may deteriorate to ataxic breathing [4]. For this breathing pattern, the respiratory rate is 0.3 Hz shown in Figure 9(d), which is not consistent with the one (0.19Hz) shown in Figure 10(d). Although the spectrogram cannot accurately estimate the breathing rate of abnormal breathing patterns but still can get a lot of useful information from it. As it can be seen form Figure 9(d), apnea occurs between 8–18 seconds and 45–55 seconds. And we can see that there are deep breathes for the first 8 seconds, 20–30seconds and 35–40seconds and shallow breathing for 30–35 seconds and 40–45seconds. This result completely coincides with that shown in Figure 10(d). Figure 10(d) shows that the measurements captured using C-band sensing technique, correlates quite closely to the measurements made by the respiration sensor.

#### 5) Kussmaul Breathing

Kussmaul breathing is deep breathing with fast, normal or slow rate. And Kussmaul breathing is often associated with severe metabolic acidosis, particularly diabetic ketoacidosis (DKA) and kidney failure [2]. Due to the periodicity of this breathing pattern, the STFT technique can be used for respiratory rate extraction. As shown in Figure 9(e), the respiratory rate approximated at 0.36 Hz is quite consistent with the respiratory rate approximated by the breathing waveform in Figure 10(e). Moreover, the normalized wireless signal is quite close to the normalized respiration sensor signal as shown in Figure 10(e).

#### 6) Sighing Breathing

Sighing breathing is a normal reaction to fatigue or to certain mild emotional states, but frequent sighs punctuating the breathing cycle may be the warning sign of hyperventilation or the early signs of depression [29]. For this type of breathing, the subject was asked to sigh frequently, which is 1.5–2 times greater than the usual tidal volume. The results of sighing breathing can be seen in Figure 10(f). From this figure, there are 5 sighs in one minute, which occur at the 10 seconds, 22 seconds, 35 seconds, 47 seconds and 58 seconds respectively. Meanwhile, Figure 9(f) accurately shows the first four sighs, and the time when these four sighs occur is consistent with the time in Figure 10(f). But the 5th sigh is missing in Figure 9(f) due to the low time resolution. Also, it is accurate to estimate the respiratory rate from Figure 9(f) where the respiratory rate is the same as shown in Figure 10(f) (both are 0.33 Hz). In this case C-band sensing technique is still capable of capturing the changes in breathing yielding consistent correlations with the respiration sensor reading as shown in Figure 10(f).

In all experiments, computation of MSE and CC as the average of three data sets for all subjects is performed between C-band sensing technique and respiratory sensor as shown in Table 3. Results present a high correlation between the C-band sensing technique and the respiratory sensor. In a word, C-band sensing technique as a non-invasive detection method is able to detect and identify different types of breathing patterns.

**TABLE 3:** The Average MSE and CC of Three Data Sets for Six Breathing Patterns From All Subjects

Types of Breathing	Subject 1		Subject 2		Subject 3	
	MSE	CC	MSE	CC	MSE	CC
Normal Breathing	0.0835	0.9050	0.0724	0.9198	0.0394	0.9465
Biot’s Breathing	0.1939	0.8270	0.0936	0.8250	0.0774	0.8376
Cheyne-Stokes Breathing	0.2306	0.8436	0.1762	0.8174	0.0846	0.8650
Kussmaul Breathing	0.2034	0.8018	0.1356	0.8350	0.0932	0.8355
Ataxic Breathing	0.1048	0.8301	0.1936	0.8217	0.0636	0.8198
Sighing Breathing	0.0739	0.8568	0.1023	0.8840	0.0521	0.8762
Types of Breathing	Subject 4		Subject 5		Subject 6	
	MSE	CC	MSE	CC	MSE	CC
Normal Breathing	0.1463	0.9164	0.1072	0.8950	0.0739	0.8674
Biot’s Breathing	0.2236	0.8610	0.1392	0.8098	0.2661	0.8374
Cheyne-Stokes Breathing	0.2176	0.8378	0.5867	0.8117	0.3565	0.8019
Kussmaul Breathing	0.3184	0.8722	0.1536	0.8550	0.0915	0.8264
Ataxic Breathing	0.1702	0.8271	0.3405	0.8205	0.5023	0.8676
Sighing Breathing	0.1248	0.8951	0.0621	0.8864	0.1064	0.9044

## VI. Conclusion

In this paper, we first demonstrated the feasibility of C-band sensing technique in capturing respiratory changes such as breathing rate, deep, and pause. We then used C-band sensing technique to detect different types of breathing patterns associated with different breathing disorders. Indeed, the experiments were conducted by all participants in professional role playing of six breathing patterns and not with real patients, yet the results are compelling that C-band sensing technique can be used as an alternative method to the standard respiratory sensor measuring the same breathing patterns. In addition, the collected data by C-band sensing technique needs to go through a series of signal processing to obtain clear respiratory waveforms. To this end, we proposed a subcarrier selection method based on scoring mechanism, a filtering method including the wavelet filter and the moving average filter, a respiration rate extraction method using the STFT technique, and a mechanism for comparing the correlation between C-band sensing technique and the contact respiratory sensor. The experimental results show that spectrograms using the STFT technique can provide adequate spectral-temporal information to understand how the breathing activity had taken place. The results also present the measurements made using C-band sensing technique, correlates quite closely to the measurements made by the respiration sensor. Therefore, we can draw a conclusion that C-band sensing technique as a non-invasive detection method is able to detect and identify different types of breathing patterns and the STFT technique is suited for detailed analysis of breathing patterns.

The use of C-band sensing technique as a sensing mechanism for respiration detection and monitoring is particularly useful owing to its unique advantages. The method can provide an effective non-contact form of use and real-time and long-term respiratory patterns monitoring in home, with no need for special hardware devices. However, there are two deficiencies in this research. One disadvantage is that currently the system is for a single subject application, the other is that the experiments were not performed on real patients. Therefore, on the one hand, future work would be extended to multiple subjects and more advanced algorithms would be applied. When there are multiple subjects in the same room, the breath of each person can be detected by C-band sensing technique, but these subjects’ breath is mixed together, which requires more advanced algorithms to distinguish each subject’s breath. On the other hand, in the future work involving real patient experiments would be conducted as well as algorithms would be used to classify corresponding breathing disorders to its appropriate classes.
